# MicroRNA-185 and 342 Inhibit Tumorigenicity and Induce Apoptosis through Blockade of the SREBP Metabolic Pathway in Prostate Cancer Cells

**DOI:** 10.1371/journal.pone.0070987

**Published:** 2013-08-09

**Authors:** Xiangyan Li, Yi-Ting Chen, Sajni Josson, Nishit K. Mukhopadhyay, Jayoung Kim, Michael R. Freeman, Wen-Chin Huang

**Affiliations:** 1 Uro-Oncology Research Program, Department of Medicine, Samuel Oschin Comprehensive Cancer Institute, Cedars-Sinai Medical Center, Los Angeles, California, United States of America; 2 Cancer Biology Program, Departments of Surgery, Medicine and Biomedical Sciences, Samuel Oschin Comprehensive Cancer Institute, Cedars-Sinai Medical Center, Los Angeles, California, United States of America; Roswell Park Cancer Institute, United States of America

## Abstract

MicroRNA (miRNA or miR) inhibition of oncogenic related pathways has been shown to be a promising therapeutic approach for cancer. Aberrant lipid and cholesterol metabolism is involved in prostate cancer development and progression to end-stage disease. We recently demonstrated that a key transcription factor for lipogenesis, sterol regulatory element-binding protein-1 (SREBP-1), induced fatty acid and lipid accumulation and androgen receptor (AR) transcriptional activity, and also promoted prostate cancer cell growth and castration resistance. SREBP-1 was overexpressed in human prostate cancer and castration-resistant patient specimens. These experimental and clinical results indicate that SREBP-1 is a potential oncogenic transcription factor in prostate cancer. In this study, we identified two miRNAs, miR-185 and 342, that control lipogenesis and cholesterogenesis in prostate cancer cells by inhibiting SREBP-1 and 2 expression and down-regulating their targeted genes, including fatty acid synthase (FASN) and 3-hydroxy-3-methylglutaryl CoA reductase (HMGCR). Both miR-185 and 342 inhibited tumorigenicity, cell growth, migration and invasion in prostate cancer cell culture and xenograft models coincident with their blockade of lipogenesis and cholesterogenesis. Intrinsic miR-185 and 342 expression was significantly decreased in prostate cancer cells compared to non-cancerous epithelial cells. Restoration of miR-185 and 342 led to caspase-dependent apoptotic death in prostate cancer cells. The newly identified miRNAs, miR-185 and 342, represent a novel targeting mechanism for prostate cancer therapy.

## Introduction

MicroRNA (miRNA or miR) is a short (average 22 nt), single stranded and endogenously occurring non-coding RNA that regulates post-transcriptional gene expression by complementary base pairing at 3′ untranslated regions (UTR) of target mRNAs [1], [2]. MiRNA controls expression of an estimated one-third of human protein-coding genes involved in fundamental cellular processes, including metabolism, differentiation, growth and apoptosis [2]–[5]. MiRNA has also been shown to play key roles in disease, particularly cancer [6]–[8]. Certain miRNA species, such as miR-20a, 23b, 34a, 126, 145, 146a, 221 and 222, are aberrantly expressed in prostate cancer [9], [10]. A number of miRNAs have been demonstrated to contribute to tumor initiation, growth and lethal progression [11]–[15]. These discoveries provide a rationale for considering the efficacy of miRNA-based replacement therapies, with the goal of inhibiting oncogenes and their related pathways, or restoring tumor suppressor genes.

Sterol regulatory element-binding protein (SREBP) is a basic helix-loop-helix leucine zipper transcription factor with important metabolic roles in lipogenesis and cholesterogenesis [16], [17]. Three major SREBP isoforms have been identified, SREBP-1a, SREBP-1c and SREBP-2 [18]. SREBP-1 controls genes involved in fatty acid, lipid and cholesterol biosynthesis [16], [17], whereas SREBP-2 more specifically regulates cholesterol metabolism and homeostasis [19]. Dysregulation of SREBPs and their downstream targeted genes associated with lipogenesis and cholesterogenesis has been implicated in cancer. Examples include fatty acid synthase (FASN), a metabolic oncogene [20], [21], and 3-hydroxy-3-methylglutaryl CoA reductase (HMGCR), the rate-limiting step in cholesterol biosynthesis; both proteins have been reported to be involved in the development and progression of prostate cancer [20], [22]–[24]. Overexpression of SREBP-1 was observed in human prostate cancer specimens compared with normal/benign prostate tissues [22], and this could be mechanistically related with progression to androgen-refractory/castration-resistant disease [22], [25]. Targeting the aberrant SREBP-lipogenesis-cholesterogenesis pathway may lead to new approaches to the treatment of prostate cancer.

The role of miRNAs in SREBP-lipogenesis-cholesterogenesis in prostate cancer remains unclear. In the present study, we identified and characterized two crucial miRNAs, miR-185 and 342, as SREBP-lipogenesis-cholesterogenesis regulators in prostate cancer cells. Both miR-185 and 342 inhibited the expression of SREBP-1 and SREBP-2 and their downstream genes, FASN and HMGCR, and further decreased the levels of fatty acid and cholesterol in prostate cancer cells. In addition, both miRNAs reduced expression of androgen receptor (AR), a known growth and survival factor. Moreover, miR-185 and 342 suppressed tumorigenicity and cell growth and induced apoptosis through activation of a caspase/PARP-mediated apoptotic pathway in prostate cancer cells and mice bearing xenograft human prostate tumors. Lower levels of miR-185 and 342 were found in prostate cancer cells compared with normal/non-cancerous prostate epithelial cells. Taken together, we demonstrate for the first time that miR-185 and 342 play a tumor suppressor role via blockade of a central lipogenesis-cholesterogenesis mechanism.

## Materials and Methods

### Prostate Cancer Cell Lines, Cell Culture and Reagents

Human prostate cancer cell lines LNCaP (androgen-dependent) and C4-2B, a LNCaP lineage-derived androgen-independent subline [26], were cultured in T-medium (Life Technologies, Grand Island, NY) supplemented with 5% fetal bovine serum (FBS), 100 IU/mL of penicillin and 100 µg/mL of streptomycin. A non-cancerous human prostate epithelial cell line, RWPE-1 (ATCC, Manassas, VA), was maintained in keratinocyte medium containing bovine pituitary extract and human recombinant epidermal growth factor (Life Technologies). All cells were maintained in 5% CO_2_ at 37°C. Human miRNA precursors, miRNA inhibitors, TaqMan miRNA assay, mirVana™ miRNA isolation kit and Lipofectamine 2000 were purchased from Life Technologies. The 3′ UTR luciferase reporter DNA constructs of SREBP-1 (HmiT017704-MT05) and SREBP-2 (HmiT017705-MT05) were purchased from GeneCopoeia (Rockville, MD). CellTiter 96® AQueous One Solution Cell Proliferation Assay (mitochondrial MTS assay) and Caspase-Glo® 3/7 Assay Systems were obtained from Promega (Madison, WI).

### MiRNA Transfection

Transient transfection of miRNA precursors or inhibitors was carried out using Lipofectamine 2000 according to the manufacturer’s protocol. Human miR-185 (PM12486) and 342 (PM13066) precursors, anti-miR-185 (AM12486) and 342 (AM13066), and negative control (miR-NC; AM17110) were used for assays.

### Quantitative Real-time Reverse Transcription-polymerase Chain Reaction (qRT-PCR)

Total RNA was prepared from cells using the RNeasy Mini kit (Qiagen, Valencia, CA) and subjected to reverse transcription by SuperScript® III reverse transcriptase (Life Technologies) according to the manufacturer’s instructions. The primer sequences used for PCR analysis are listed in Table S1. A hot start at 95°C for 5 min was followed by 40 cycles at denaturation at 95°C for 15 s, annealing of the primers at 60°C for 30 s and elongation at 72°C for 30 s using ABI 7500 Fast Real-Time PCR System (Life Technologies). Data were normalized to 18S rRNA or GAPDH and represented as the average fold of three independent duplicates. To determine intrinsic miR-185 and 342 expression, miRNA was prepared from cells using the mirVana™ miRNA isolation kit (Life Technologies). Mature miRNA was quantified by the TaqMan miRNA assay (Life Technologies) in accordance with the manufacturer’s instructions. The data were normalized by RNU6B.

### Western Blot Analysis

Cell lysates were prepared from miR-185, 342, NC (miR-negative control)-transfected or non-transfected prostate cancer cells using a lysis buffer [50 mM Tris (pH 8), 150 mM NaCl, 0.02% NaN_3_, 0.1% SDS, 1% NP-40 and 0.5% sodium deoxycholate] containing 1 mM phenylmethylsulfonyl fluoride and protease inhibitor cocktail (Roche Applied Science, Indianapolis, IN). Protein concentration was determined by Bradford assay using Coomassie Plus Protein Reagent (Thermo Scientific, Rockford, IL). Western blot was performed using the Novex system (Life Technologies) as described previously [27], [28]. Primary antibodies anti-SREBP-1, SREBP-2, FASN, HMGCR, AR and β2-microglobulin (β2M) (Santa Cruz Biotechnology, Santa Cruz, CA), and secondary antibodies which were conjugated with horseradish peroxidase (GE Healthcare, Piscataway, NJ) were used. Detection of protein bands was done using Enhanced Chemiluminescence Western Blotting Detection Reagents (GE Healthcare).

### Cell Proliferation, Clonogenicity, Migration and Invasion Assays

LNCaP or C4-2B cells (6,000 cells/well) were plated on 96-well plates and transfected with miR-185, 342 or NC. Cell proliferation was measured 3 d post-transfection by MTS assay (Promega) according to the manufacturer’s instructions. For the clonogenicity assay, LNCaP or C4-2B cells transfected with miR-185, 342 or NC were seeded on 6-well plates (500 cells/well). Cells were cultured at 37°C with 5% CO_2_ for 14 d. Colonies were fixed with formalin and stained with crystal violet [27]. The numbers of developed colonies were counted and analyzed for significant differences. *In vitro* cell migration or invasion was determined in Boyden chambers pre-coated with collagen I (Sigma-Aldrich, St. Louis, MO; for migration assay) or growth factor-depleted Matrigel matrix (BD Bioscience, San Jose, CA; for invasion assay). Cells (1.5×10^5^ cells/well) transfected with miR-185, 342 or NC were seeded into the inside of the pre-coated upper chambers. After incubation at 37°C with 5% CO_2_ for 48 h, the numbers of migrated or invading cells were measured by a crystal violet staining method [27].

### Fatty Acid and Cholesterol Quantification

The amounts of long chain fatty acids and cholesterols were determined using the Free Fatty Acid Quantification Kit and Cholesterol/Cholesteryl Ester Detection Kit (Abcam, Cambridge, MA) in cells transfected with miR-185, 342 or NC. The amounts of fatty acids and cholesterols were normalized by total cell numbers. The relative fatty acid or cholesterol (%) was assigned as 100% in NC cells.

### Apoptosis Assays

For Annexin V staining, a fluorescence-activated prostate cancer cell sorting analysis was done 72 h post-transfection of miRNAs using the Annexin V-FITC Apoptosis Detection Kit I (BD Bioscience) and a FACScan flow cytometer with CellQuest software (Becton Dickinson Labware, Lincoln Park, NJ). For the caspase activity assay, cells transfected with miRNAs for 24 h were measured for caspase 3/7 enzymatic activities by Caspase-Glo® 3/7 Assay Systems (Promega). Data were normalized by total proteins. For caspase-Western blot analysis, whole cell lysates were prepared from cells transfected with miRNAs for 72 h and subjected to Western blot. Anti-caspase 9, 3 and PARP primary antibodies (Cell Signaling Technology, Beverly, MA) were used.

### Ethics Statement

All the animal experiments were carried out in strict accordance with the recommendations in the Guide for the Care and Use of Laboratory Animals of the National Institutes of Health. The protocol was approved by the Institutional Animal Care and Use Committee of Cedars-Sinai Medical Center (IACUC Protocol #004252) and was followed for the mouse studies.

### *In vivo* Animal Experiments

To examine the anti-tumor efficacy of miR-185 and 342 *in vivo*, six-week-old male athymic nude mice (Harlan Laboratories, Placentia, CA) were inoculated subcutaneously with 2×10^6^ C4-2B cells per mouse. The tumor burden was monitored by tumor volume (using the formula V = 4/3π × (d/2)^2^ × D/2, where d is the minor tumor axis and D is the major tumor axis). After six weeks inoculation, 100 pmole of miRNA complexed with 3 µL of Lipofetamine 2000 in 50 µL of PBS was delivered intratumorally every 3 d for a 21-d treatment. The dose and schedule of miRNA injection were modified by a previous report [29]. Upon termination of the animal study, tumor tissues were harvested from the euthanized mice and fixed in 10% formalin, dehydrated in ethanol, embedded in paraffin and sectioned for slides. The blank tissue slides were subjected to immunohistochemical staining (IHC) [30] using anti-SREBP-1, SREBP-2, FASN, HMGCR, AR, PSA, Ki67 and cleaved PARP primary antibodies. For quantification of Ki67 (cell proliferation) and cleaved PARP (apoptosis) expression, 100 tumor cells at 5 randomly selected areas were counted and positively staining cells were recorded. Additionally, partial fresh tumor tissues were collected to determine the expression of miR-185 or 342 by qRT-PCR. miRNA was prepared from tumor tissues using the mirVana™ miRNA isolation kit.

### Statistical Analysis

Statistical analysis was performed as described previously [31]. Student’s *t*-test and two-tailed distribution were applied for the analysis of statistical significance.

## Results

### MiR-185 and 342 Inhibit Expression of SREBPs and their Downstream Genes in Prostate Cancer Cells

**Figure 1:**
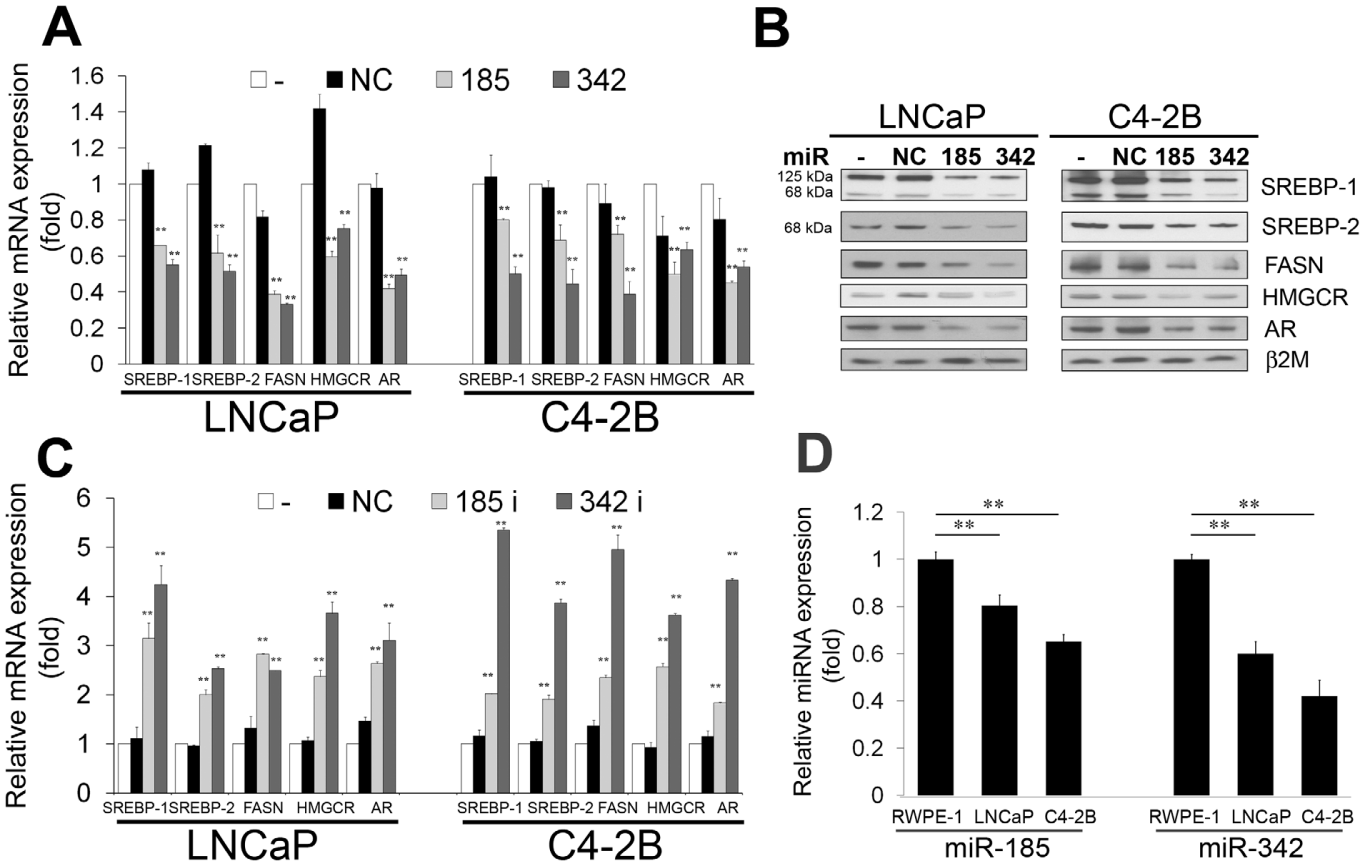
MiR-185 and 342 inhibition of SREBP mRNA and protein and expression patterns of miR-185 and 342 in prostate cancer cells. **A,** Both miR-185 and 342 inhibited mRNA expression of SREBP-1, SREBP-2, FASN, HMGCR and AR in LNCaP and C4-2B prostate cancer cells determined by qRT-PCR. -: non-transfected; NC: miR-negative control. The relative mRNA expression (fold) was assigned as 1.0 in non-transfected cells. Data were normalized to 18S rRNA and represent the mean ± SD of three independent duplicate experiments. **, *P* < 0.005 significant differences from NC. **B,** MiR-185 and 342 inhibited precursor (125 kDa) and mature (68 kDa) forms of SREBP-1 and SREBP-2, FASN, HMGCR and AR expression in LNCaP and C4-2B cells assayed by Western blot. β2-microglobulin (β2M) was used as a loading control. **C,** MiR-185 and 342 inhibitors (antisense oligonucleotides against miR-185 and 342) increased SREBP-1, SREBP-2, FASN, HMGCR and AR expression in LNCaP and C4-2B cells determined by qRT-PCR. The relative mRNA expression (fold) was assigned as 1.0 in non-transfected cells. Data were normalized to 18S rRNA and represent the mean ± SD of three independent duplicate experiments. **, *P* < 0.005 significant differences from NC. **D,** Expression of intrinsic miR-185 and 342 in RWPE-1, LNCaP and C4-2B cells. qRT-PCR results showed that relative expression of miR-185 and 342 was significantly decreased in prostate cancer cells compared with normal/non-cancerous RWPE-1. Lower expression of both miRNAs was observed in aggressive C4-2B compared with LNCaP cells. The relative miRNA expression (fold) was assigned as 1.0 in RWPE-1 cells. **, *P* < 0.005 significant differences from RWPE-1. Data were normalized to RNU6B control and represent the mean ± SD of three independent experiments performed in quadruplicate.

To investigate whether miRNAs regulate the SREBP-lipogenesis-cholesterogenesis metabolic pathway in prostate cancer cells, we first used TargetScanHuman 6.2 online software (http://www.targetscan.org/) to predict if one or more miRNAs target both SREBP-1 and SREBP-2, two key transcription factors that regulate fatty acid, lipid and cholesterol biosynthesis and homeostasis. Two miRNAs, miR-185 and 342, were retrieved that potentially co-targeted 3′ UTRs of SREBP-1 and SREBP-2 mRNAs (Fig. S1A). To further verify if miR-185 and 342 directly bind with 3′ UTRs of SREBP-1 and SREBP-2, we performed 3′ UTR luciferase reporter assay and found that the relative 3′ UTR luciferase activities of both SREBP-1 and SREBP-2 were significantly decreased in miR-185 and 342 transfected prostate cancer cells (Fig. S1B). The results confirm that SREBP-1 and SREBP-2 mRNAs are direct targets of miR-185 and 342. To validate whether these two miRNAs control the SREBP-lipogenesis-cholesterogenesis pathway in prostate cancer cells, we performed qRT-PCR, Western blot, and fatty acid and cholesterol quantification analyses. Both miR-185 and 342 inhibited the expression of mRNAs (Fig. 1A) as well as precursor (125 kDa) and mature (68 kDa) proteins (Fig. 1B) of SREBP-1 and SREBP-2 in LNCaP and C4-2B prostate cancer cells. Expression of FASN [32] and HMGCR [33], [34], which are SREBP downstream regulated genes, was decreased by miR-185 and 342 (Fig. 1A and 1B). MiR-185 and 342 also reduced AR mRNA and protein expression in prostate cancer cells (Fig. 1A and 1B). Because FASN and HMGCR are important enzymes for *de novo* synthesis of fatty acid and cholesterol individually, we subsequently examined the levels of fatty acid and cholesterol in cells. As shown in Table 1, the amounts of intracellular fatty acid and cholesterol were significantly decreased in miR-185 and 342-transfected LNCaP and C4-2B cells compared with the control groups. To further test the specificity of miR-185 and 342 for SREBP-lipogenesis-cholesterogenesis, antisense oligonucleotides against miR-185 and 342 were used as miR-185 and 342 inhibitors. By blocking endogenous miR-185 and 342 in prostate cancer cells, both miR-185 and 342 inhibitors increased SREBP-1, SREBP-2 and their downstream gene expression (Fig. 1C). These results suggest that miR-185 and 342 inhibited SREBP signaling through reduction of SREBP mRNA and protein and decreased the levels of fatty acid and cholesterol in prostate cancer cells.

**Table 1:** MiR-185 and 342 decreased the amounts of fatty acid and cholesterol in prostate cancer cells.

	LNCaP		C4-2B	
	Relative fatty acid (%)	Relative cholesterol (%)	Relative fatty acid (%)	Relative cholesterol (%)
NC	100±0.2	100±3.2	100±2.5	100±3.3
185	89.2±1.4*	86.1±3.5*	90.8±0.2*	89.6±3.2*
342	82.2±4.3**	85.8±1.6**	87.7±1.5**	88.0±3.7*

* *P*<0.05; ** *P*<0.005.

Next, we determined the expression of intrinsic miR-185 and 342 in various cell lines with clinical relevance, including a human normal/non-cancerous prostate epithelial cell line, RWPE-1, and LNCaP (androgen-dependent) and C4-2B (androgen-independent) prostate cancer cells. MiRNA was prepared from these cells. Relative expression of both miR-185 and 342 was significantly decreased in cancer cells compared to non-cancerous RWPE-1 (Fig. 1D) as assayed by qRT-PCR. Additionally, lower miR-185 and 342 expression was observed in the more aggressive C4-2B line in comparison with LNCaP. Furthermore, we examined the expression of SREBPs, FASN and HMGCR to correlate with intrinsic miR-185 and 342 in these cell lines. Relative expression of SREBP-1, SREBP-2, FASN and HMGCR was higher in LNCaP and C4-2B than in RWPE-1 cells (Fig. S2). These data indicate that miR-185 and 342 are negative regulators for SREBP signaling in prostate cancer cells.

### MiR-185 and 342 Suppress Cell Proliferation, Clonogenicity, Migration and Invasion in Prostate Cancer Cells

**Figure 2:**
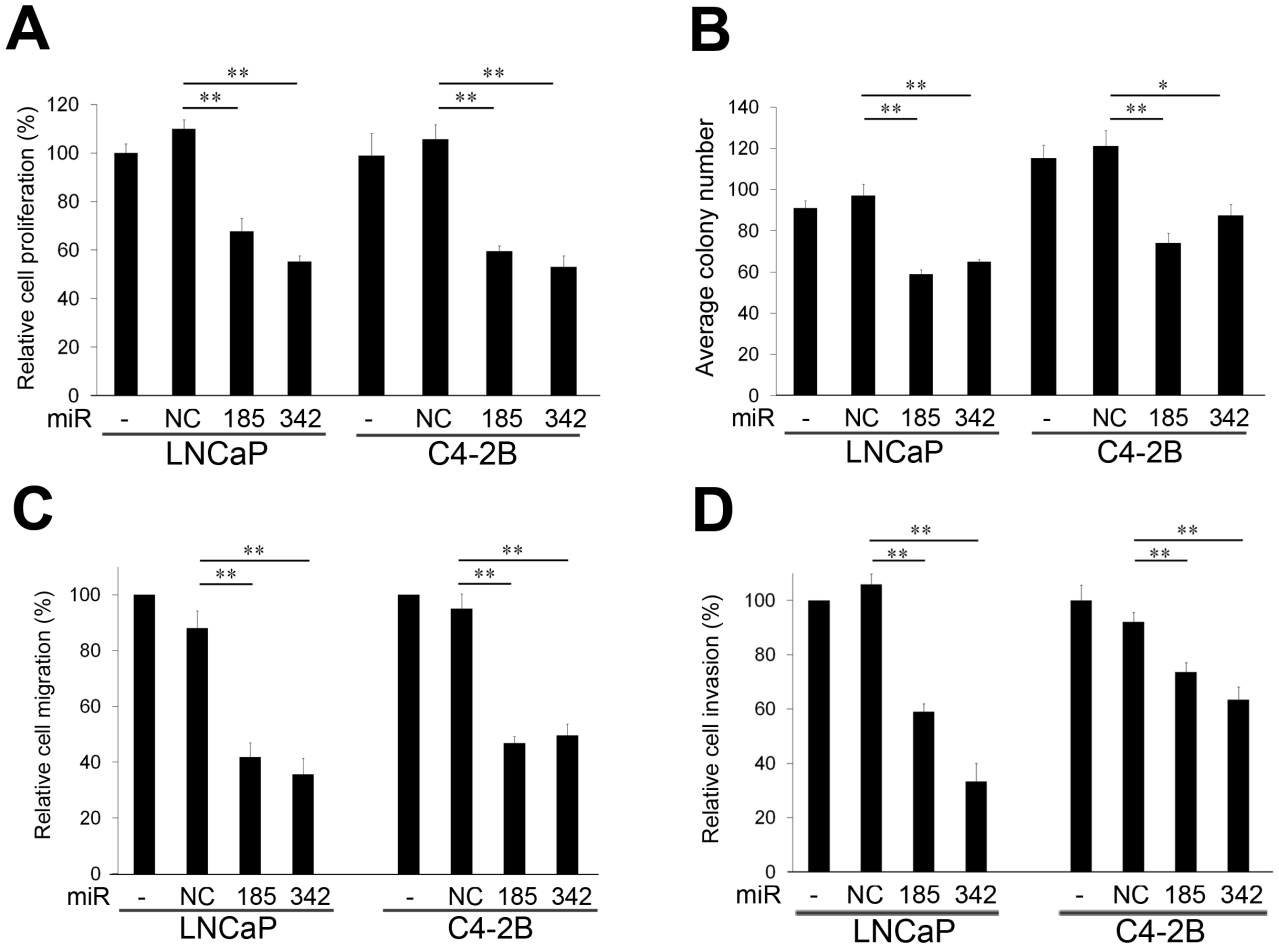
MiR-185 and 342 suppress cell proliferation, clonogenicity, migration and invasion. **A,** MiR-185 and 342 inhibited cell proliferation in LNCaP and C4-2B cells compared with non-transfected (−) and miR-negative control (NC) transfected cells 3 d following miRNA transfection. The relative cell proliferation (%) was assigned as 100% in non-transfected (−) cells. **, *P* < 0.005 significant differences from NC. Data represent the mean ± SD of two independent quadruplicate experiments. **B,** MiR-185 and 342 suppressed colony formation in LNCaP and C4-2B cells compared with the control groups after 14 d miRNA transfection. *, *P* < 0.05 and **, *P* < 0.005 significant differences from NC. Data represent the mean ± SD of two independent triplicate experiments. **C,** Cell migration and **D,** invasion were significantly decreased by miR-185 and 342 in LNCaP and C4-2B cells compared with the control groups. **, *P* < 0.005 significant differences from NC. Data represent the mean ± SD of two independent quadruplicate experiments.

To assess the potential for biological consequences elicited by miR-185 and 342, we re-expressed miR-185 and 342 in LNCaP and C4-2B cells and performed a series of functional assays relevant to tumorigenicity and cancer progression. When LNCaP and C4-2B cells were transfected with miR-185 and 342, proliferation of both cell types was inhibited in comparison with miR-NC and non-transfected cells (Fig. 2A). Both miR-185 and 342 also decreased clonogenicity compared with the control groups (Fig. 2B). One of the hallmarks of progressive and metastatic cells is their ability to invade surrounding tissues and migrate efficiently [35]. MiR-185 and 342 significantly inhibited *in vitro* migration (Fig. 2C) and invasion (Fig. 2D) in LNCaP and C4-2B cells. Conversely, by blocking miR-185 and 342 in prostate cancer cells, miR-185 and 342 inhibitors increased cell proliferation, colony formation, migration and invasion (Fig. S3). These data suggest that both miR-185 and 342 suppress pathways relevant to tumorigenicity and cancer progression.

### MiR-185 and 342 Induce caspase-dependent Apoptotic Death in Prostate Cancer Cells

**Figure 3:**
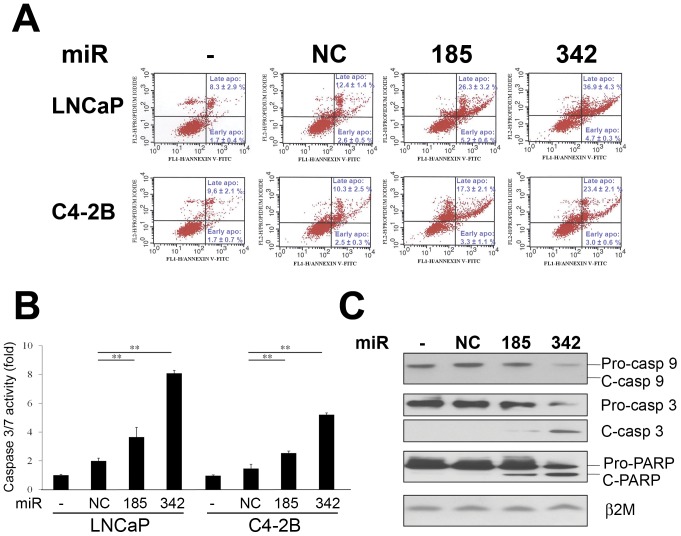
MiR-185 and 342 induce caspase-dependent apoptotic death. **A,** An Annexin V-FITC/PI staining apoptotic assay and flow cytometry were performed in LNCaP and C4-2B cells transfected with miRNAs. Both miR-185 and 342 increased the apoptotic cell fractions (both early and late apoptotic cell fractions; *P*<0.005) compared with the control groups. **B,** Caspase 3/7 activities were significantly increased by miR-185 and 342 in LNCaP and C4-2B cells. Caspase 3/7 activities (fold) were assigned as 1.0 in non-transfected (−) cells. **, *P* < 0.005 significant differences from NC. Data represent the mean ± SD of two independent triplicate experiments. **C,** MiR-185 and 342 decreased pro-caspase 9, 3 and PARP, and activated cleaved caspase 3 and PARP expression in LNCaP cells as assayed by Western blot. β2M was used as a loading control. C: cleaved.

To determine if miR-185 and 342 induce apoptosis in prostate cancer cells, Annexin V-FITC/propidium iodide (PI) staining measurement, caspase activity assay and Western blot of caspase and PARP expression were conducted. The results of Annexin V-FITC/PI staining and flow cytometric analysis revealed that miR-185 and 342 increased the apoptotic cell fractions (both early and late apoptotic cell fractions, *P*<0.005) in LNCaP and C4-2B cells compared with the control groups (Fig. 3A). Caspase 3/7 activities were also significantly induced by miR-185 and 342 in LNCaP and C4-2B cells (Fig. 3B). Furthermore, Western blot results showed that pro-caspase 9 and 3 were decreased by miR-185 and 342 (Fig. 3C). Also, cleaved caspase-3 and PARP, a downstream factor of caspases, were detected in miR-185 and 342 transfected cells (Fig. 3C). These results suggest that miR-185 and 342 induce prostate cancer cell death through activation of a caspase-dependent apoptotic mechanism.

### Intratumoral Delivery of miR-185 and 342 Leads to Regression of Prostate Tumors in a Mouse Xenograft Model

**Figure 4:**
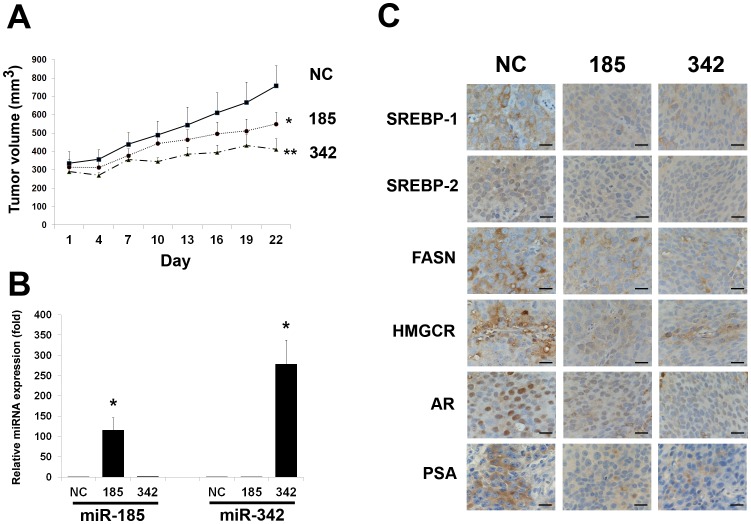
Intratumoral delivery of miR-185 and 342 leads to regression of prostate tumors in a mouse xenograft model. **A,** Subcutaneous C4-2B tumor growth was assayed by tumor volume after intratumoral delivery of miR-185, 342 or NC every 3 d for a 21-d treatment in mice. Both miR-185 and 342 significantly inhibited the growth of C4-2B tumors compared with NC-treated tumors. *, *P*<0.05; **, *P*<0.005 significant differences from NC-treated tumors (N = 5 for each group). **B,** The relative expression of miR-185 or 342 in C4-2B tumors. qRT-PCR results showed that the relative miR-185 or 342 levels were greatly increased in the subcutaneous C4-2B tumors injected with miR-185 or 342 compared with the control tumors. The relative miRNA expression (fold) was assigned as 1.0 in NC. *, *P* < 0.05 significant differences from NC. Data were normalized to RNU6B and represent the mean ± SD. **C,** IHC results showed that down-regulation of SREBP-1, SREBP-2, FASN, HMGCR, AR and PSA was observed in the miR-185 and 342-treated subcutaneous C4-2B tumors in comparison with the NC-treated tumors. Scale bars = 25 µm.

**Figure 5:**
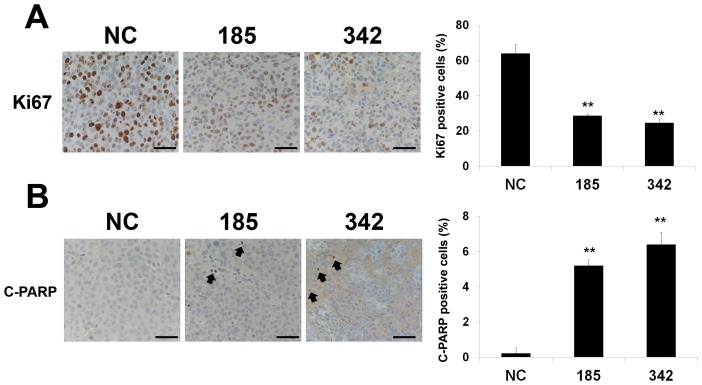
MiR-185 and 342 inhibit cell proliferation and induce apoptosis in subcutaneous xenografts. Quantification of **A,** Ki67 (cell proliferation) and **B,** cleaved PARP (C-PARP; apoptosis) positive cells in subcutaneous C4-2B tumor specimens collected from the miR-NC, 185 and 342 treated groups. One hundred cells at 5 randomly selected areas were counted and positively staining cells were recorded. **, *P* < 0.005 significant differences from the control NC group. Data represent the mean ± SD. Arrowheads indicate the C-PARP positive cells. Scale bars = 100 µm.

Because the *in vitro* data demonstrated anti-tumorigenic and apoptotic roles of miR-185 and 342 in prostate cancer cells, we subsequently examined the therapeutic potential of miR-185 and 342 using a mouse subcutaneous prostate tumor xenograft model. After a 21-d treatment by intratumoral delivery every 3 d, both miR-185 and 342 significantly reduced subcutaneous C4-2B tumor burden compared with the control group (Fig. 4A). To correlate the therapeutic response with the delivery of miRNA, miRNA was isolated from fresh C4-2B tumors and the levels of miR-185 and 342 were assessed by qRT-PCR. Tumors injected with miR-185 or 342 contained approximately 116±30-fold (miR-185, *P* < 0.05) or 278±59-fold (miR-342, *P* < 0.05) higher than the control tumors (Fig. 4B). These findings suggest that the inhibition of subcutaneous C4-2B growth was due to miR-185 or 342 treatment. Consistent with previous *in vitro* Western blot results (Fig. 1B), IHC data showed that the miR-185 or 342 treated groups had decreased SREBP-1, SREBP-2, FASN, HMGCR, AR and PSA, which is an AR downstream target gene, expression compared with the control tumors (Fig. 4C). Furthermore, to determine the effects of miR-185 and 342 on cell proliferation and apoptosis *in vivo*, we performed the biomarker Ki67 and cleaved PARP staining in the C4-2 tumors. As shown in Fig. 5A and 5B, markedly decreased cell proliferation (Ki67) and increased apoptotic death (cleaved PARP) of C4-2B tumors were observed in both miR-185 and 342 treated tumor specimens compared with the control group. The *in vivo* results indicate that direct application of miR-185 and 342 to prostatic tumors can provide a therapeutic benefit in a model prostate cancer system.

## Discussion

Aberrant lipid and cholesterol anabolism is strongly linked with prostate cancer [36], [37]. Up-regulation of lipogenesis and cholesterogenesis in cancer cells is associated with increased need for cell membrane components and activation of lipid raft-related intercellular signaling transduction during uncontrolled cell proliferation and division as well as cancer development and progression [24], [38]–[41]. Blockade of abnormal lipogenesis and cholesterogenesis provides a promising therapeutic approach for prevention or treatment of prostatic malignancy. MiRNA has been reported to regulate multiple important biological processes including metabolism [3], [5] and is of potential use in cancer therapy [42]–[44]. However, how miRNA mediates aberrant fat metabolism and homeostasis in prostate cancer cells remains unclear. In this study, we identify two miRNAs that play an important role in the regulation of lipogenesis and cholesterogenesis in prostate cancer cells. MiR-185 and 342 inhibited fatty acid and cholesterol biosynthesis through down-regulation of key lipogenic and cholesterogenic transcription factors, SREBP-1 and SREBP-2, and their downstream regulated genes including FASN and HMGCR. SREBP-1 and FASN have been shown to be a potentially oncogenic transcription factor [22] and a metabolic oncogene [20], [21], respectively. MiR-185 and 342 reduced cell proliferation, clonogenicity, migration and invasion, and induced caspase-dependent apoptosis in prostate cancer cells. These data suggest that miR-185 and 342 play a tumor-suppressive role by inhibiting SREBP-1 and SREBP-2 expression, and thereby reprogramming lipogenesis and cholesterogenesis.

A number of miRNAs have been identified to be linked to prostate cancer development and progression to lethal disease. MiR-20a was reported to regulate cell proliferation and progression through inhibition of gap junction protein connexin 43 expression in prostate cancer [45]. Diminished expression of miR-143 and 145 was found in prostate cancer patients with bone metastasis [46]. Also, both miR-143 and 145 mediated epithelial-mesenchymal transition (EMT) and suppressed the metastatic capability of PC3 prostate cancer cells *in vitro* and *in vivo* [46]. MiR-203 down-regulated a cohort of metastasis-related genes and prevented bone metastasis in prostate cancer [47]. By blocking CD44 expression, miR-34a inhibited migration and invasion in prostate cancer stem cells [48]. MiR-let-7c decreased AR expression and activity in prostate cancer cells by targeting an oncogenic transcription factor, c-Myc [49]. Analysis of clinical tumor samples collected from bony metastatic castration resistant prostate cancer (CRPC) patients revealed that aberrant miR-23b/27b expression could be involved in progression to castration resistance [50]. In addition, miR-221 and 222 have been demonstrated to control the development of CRPC [12]. In the present study, we discovered two new lipid and cholesterol anabolism regulated miRNAs, miR-185 and 342, that blocked the SREBP-lipogenesis-cholesterogenesis, decreased AR expression, inhibited tumorigenicity and induced apoptotic death in prostate cancer cells. A combination of miR-185 and 342 did not show the significantly additive or synergistic effect on gene expression, growth, migration and invasion compared to single miRNA in prostate cancer cells (data not shown). Moreover, expression of intrinsic miR-185 or 342 was significantly low in prostate cancer cells compared to normal/non-cancerous epithelial cells. Further investigation of the expression profiles of miR-185 and 342 in human prostate tumor specimens is warranted. Collectively, these experimental and clinical studies suggest that miRNAs play critical and significant roles in prostate cancer development and progression.

AR is an important androgenic hormone-activated transcription factor and a growth and survival regulator for androgen-dependent organs during normal development and neoplastic progression. Increases of AR expression and activity have been well documented to be associated with prostate cancer development and CRPC [51]–[53]. Silencing AR and interrupting AR regulated signaling pathways are heavily investigated avenues for prostate cancer therapy. Many strategies have been attempted targeting AR through the inhibition of AR gene expression [30], [54] or interruption of the interaction between AR and its co-factors and their downstream functions [52], [55], [56] in prostate cancer cells. In this study, we revealed that miR-185 and 342 suppressed AR expression in cell culture (Fig. 1A and 1B) as well as in subcutaneous C4-2B tumors (Fig. 4C). The inhibitory mechanism of AR expression by miR-185 and 342 is likely to be down-regulation of SREBP-1. We previously showed that SREBP-1 regulates AR gene expression by binding an SREBP-1 *cis*-acting element located in the 5′ flanking AR promoter region [30]. Additionally, miR-185 has been demonstrated to directly bind with the 3′ UTR of AR mRNA to further decrease AR expression [57]. Analysis of the 3′ UTR of AR failed to find a binding site for miR-342 ([57] and data not shown), suggesting that suppression of AR by miR-342 may be not through the typical miRNA mediated mechanism. Rather, these results suggest a novel mechanism whereby miR-185 and 342 inhibit AR expression through the transcriptional regulation of SREBP in prostate cancer cells. This finding also could be exploited for therapeutic application by co-targeting the lipogenic and cholesterogenic metabolic pathways and AR signaling using miR-185 and 342.

In summary, our study demonstrates for the first time that: 1) MiR-185 and 342 inhibit the expression of SREBP-1 and SREBP-2 as well as their downstream regulated genes, and reprogram lipogenesis and cholesterogenesis in prostate cancer cells. 2) Intrinsic miR-185 and 342 expression is significantly decreased in prostate cancer cells compared to non-cancerous prostate epithelial cells. Furthermore, lower miR-185 and 342 expression is found in aggressive androgen-independent C4-2B in comparison with androgen-responsive LNCaP cells. Additional studies are warranted to define the regulatory mechanisms involving miR-185 and 342 in prostate cancer cells. 3) Re-expression of miR-185 or 342 suppresses tumorigenicity and cell proliferation, and induces apoptotic cell death *in vitro* and *in vivo*. It suggests that miR-185 and 342 play a tumor-suppressive role in prostate cancer. 4) While miR-185 and 342 mediate lipogenesis and cholesterogenesis, these two miRNAs also inhibit AR mRNA and protein expression. Taken together, miR-185 and 342 directly or indirectly regulate a cohort of genes with significant biological roles in lipid and cholesterol anabolism and homeostasis, cell proliferation and progression in prostate cancer cells. These two small non-coding RNAs therefore provide potential therapeutic agents for treatment of prostate cancer malignancy.

## Supporting Information

Figure S1 **SREBP-1 and SREBP-2 mRNAs are direct targets of miR-185 and 342.** **A,** Schematic representation of the relative positions of putative miR-185 and 342 target sites in SREBP-1 and SREBP-2 mRNA 3′ UTRs. **B,** 3′ UTR luciferase reporter assay. The relative 3′ UTR luciferase activities of both SREBP-1 and SREBP-2 were significantly decreased in miR-185 and 342 transfected LNCaP cells compared to miR-NC transfected cells. **, *P* < 0.005 significant differences from NC. NC: negative control.
(PDF)

Figure S2 **Expression of SREBP-1, SREBP-2, FASN and HMGCR in RWPE-1, LNCaP and C4-2B cells.** The qRT-PCR results showed that the relative expression of SREBP-1, SREBP-2, FASN and HMGCR was significantly increased in prostate cancer cells compared to normal/non-cancerous RWPE-1. The relative mRNA expression (fold) was assigned as 1.0 in RWPE-1 cells. **, *P* < 0.005 significant differences from RWPE-1. Data represent the mean ± SD of two independent experiments performed in quadruplicate.
(PDF)

Figure S3 **MiR-185 and 342 inhibitors induce cell proliferation, colony formation, migration and invasion.** **A,** MiR-185 and 342 inhibitors induced cell proliferation in LNCaP cells compared to miR-negative control (NC) transfected cells 3 d following miRNA inhibitor transfection. The relative cell proliferation (%) was assigned as 100% in NC. **, *P* < 0.005 significant differences from NC. **B,** MiR-185 and 342 inhibitors increased colony formation in LNCaP cells compared to NC after 14 d miRNA transfection. **, *P* < 0.005 significant differences from NC. **C,** Cell migration and **D,** invasion were significantly induced by miR-185 and 342 inhibitors in LNCaP cells compared to NC. The relative cell migration or invasion (%) was assigned as 100% in NC. **, *P* < 0.005 significant differences from NC. Data represent the mean ± SD of two independent quadruplicate experiments.
(PDF)

Table S1 **The sequences of primers used for qPCR.**
(PDF)
