# Supplementary figures and images for: MicroRNA-185 and 342 Inhibit Tumorigenicity and Induce Apoptosis through Blockade of the SREBP Metabolic Pathway in Prostate Cancer Cells
Source: PLoS One. 2013 Aug 9;8(8):e70987. doi: 10.1371/journal.pone.0070987 (PMC3739799; doi:10.1371/journal.pone.0070987)

Figure S1

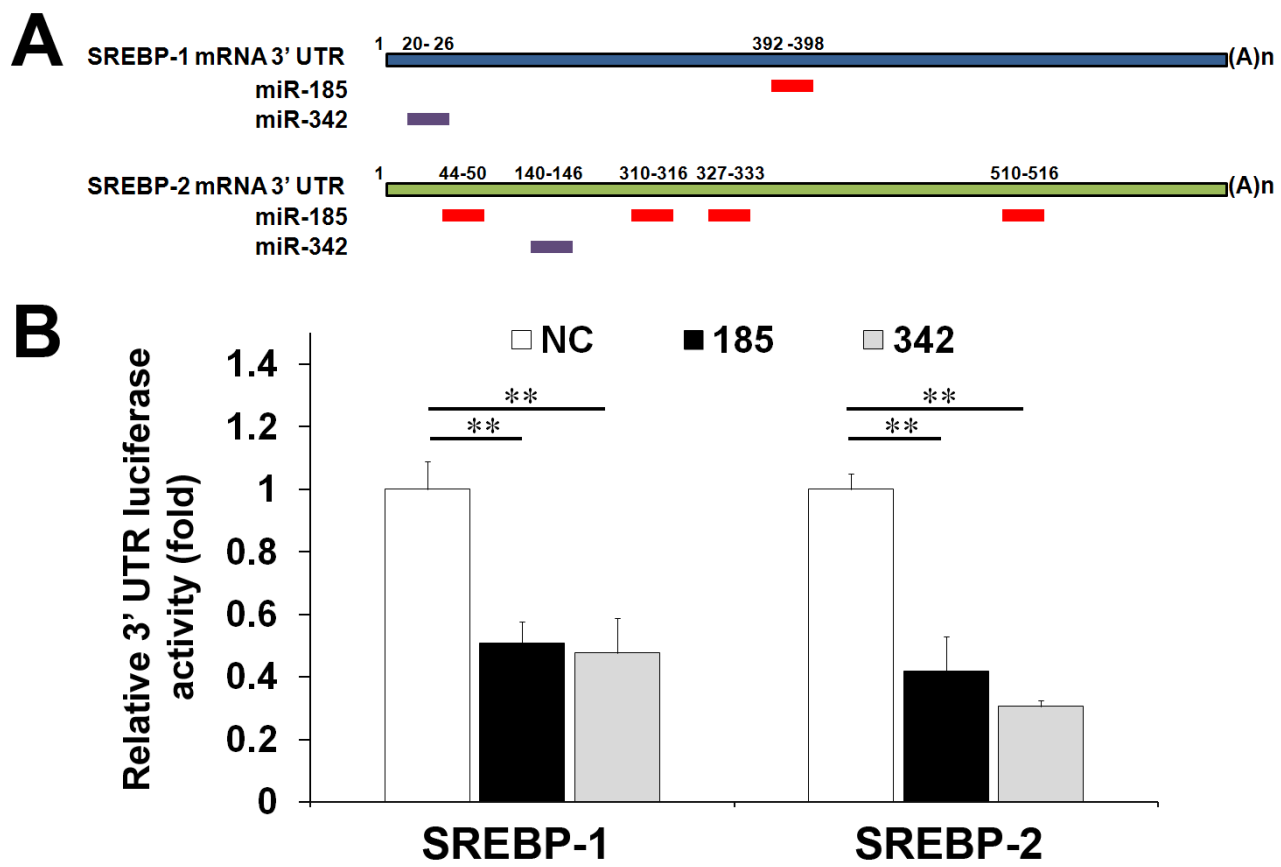

Supplement: Figure S1 — SREBP-1 and SREBP-2 mRNAs are direct targets of miR-185 and 342. A, Schematic representation of the relative positions of putative miR-185 and 342 target sites in SREBP-1 and SREBP-2 mRNA 3′ UTRs. B, 3′ UTR luciferase reporter assay. The relative 3′ UTR luciferase activities of both SREBP-1 and SREBP-2 were significantly decreased in miR-185 and 342 transfected LNCaP cells compared to miR-NC transfected cells. **, P < 0.005 significant differences from NC. NC: negative control. (PDF) [file pone.0070987.s001.pdf]

**Figure S2**

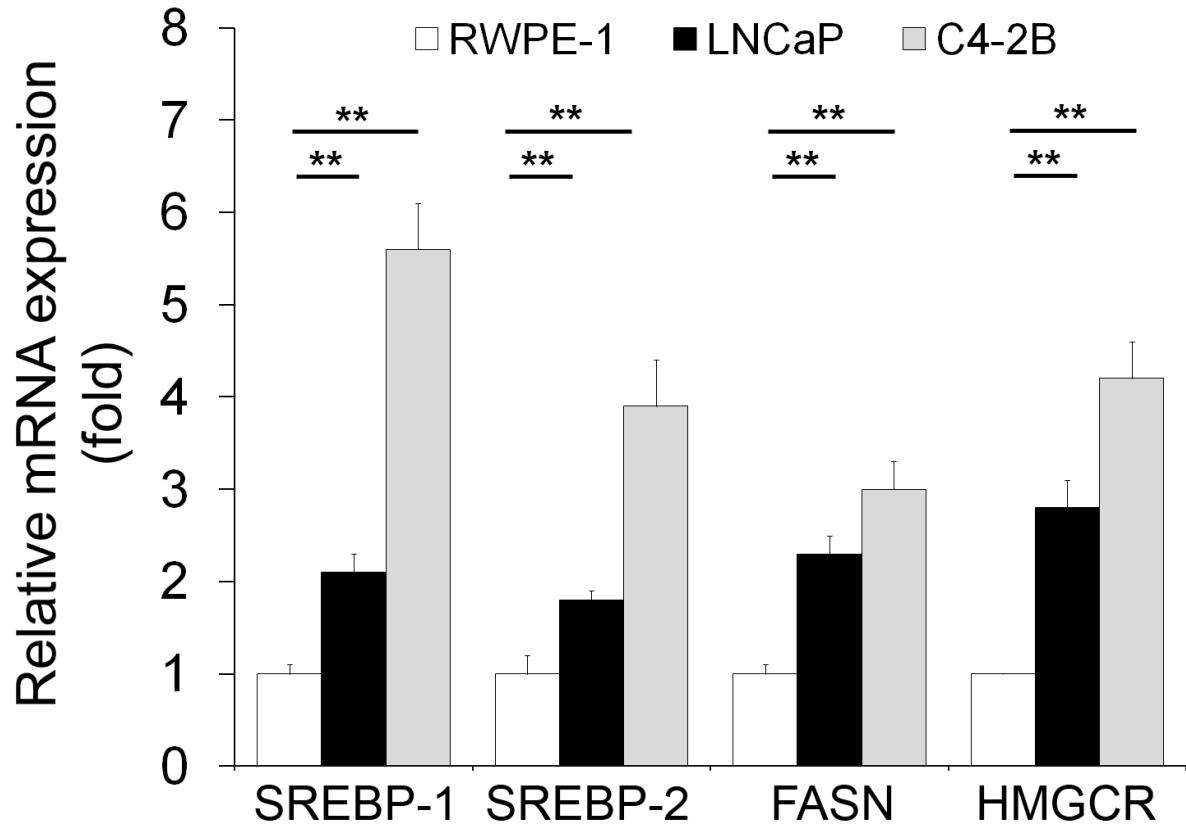

Supplement: Figure S2 — Expression of SREBP-1, SREBP-2, FASN and HMGCR in RWPE-1, LNCaP and C4-2B cells. The qRT-PCR results showed that the relative expression of SREBP-1, SREBP-2, FASN and HMGCR was significantly increased in prostate cancer cells compared to normal/non-cancerous RWPE-1. The relative mRNA expression (fold) was assigned as 1.0 in RWPE-1 cells. **, P < 0.005 significant differences from RWPE-1. Data represent the mean ± SD of two independent experiments performed in quadruplicate. (PDF) [file pone.0070987.s002.pdf]

**Figure S3**

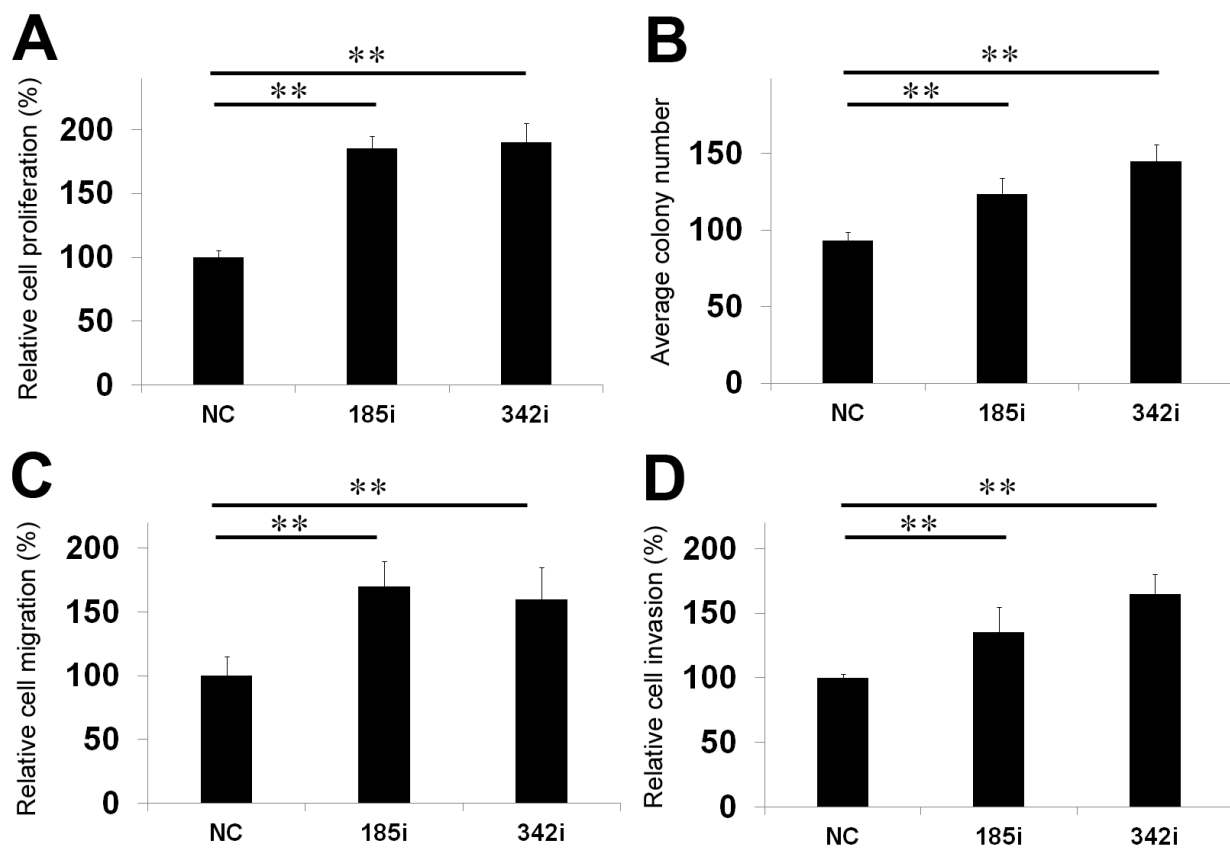

Supplement: Figure S3 — MiR-185 and 342 inhibitors induce cell proliferation, colony formation, migration and invasion. A, MiR-185 and 342 inhibitors induced cell proliferation in LNCaP cells compared to miR-negative control (NC) transfected cells 3 d following miRNA inhibitor transfection. The relative cell proliferation (%) was assigned as 100% in NC. **, P < 0.005 significant differences from NC. B, MiR-185 and 342 inhibitors increased colony formation in LNCaP cells compared to NC after 14 d miRNA transfection. **, P < 0.005 significant differences from NC. C, Cell migration and D, invasion were significantly induced by miR-185 and 342 inhibitors in LNCaP cells compared to NC. The relative cell migration or invasion (%) was assigned as 100% in NC. **, P < 0.005 significant differences from NC. Data represent the mean ± SD of two independent quadruplicate experiments. (PDF) [file pone.0070987.s003.pdf]
