# Supplementary material for: MicroRNA-185 and 342 Inhibit Tumorigenicity and Induce Apoptosis through Blockade of the SREBP Metabolic Pathway in Prostate Cancer Cells
Source: PLoS One. 2013 Aug 9;8(8):e70987. doi: 10.1371/journal.pone.0070987 (PMC3739799; doi:10.1371/journal.pone.0070987)
Supplement: Table S1 — The sequences of primers used for qPCR. (PDF) [file pone.0070987.s004.pdf]

Supplementary Table S1. The sequences of primers used for qPCR.

Gene		Sequence (5' to 3')
SREBP-1a	Forward	GGAGGGGTAGGGCCAACGGCCT
	Reverse	CATGTCTTCGAAAGTGCAATCC
SREBP-2	Forward	CCCCTGACTTCCCTGCTGCA
	Reverse	GCGCGAGTGTGGCCGGATC
FASN	Forward	CGGTACGCGACGGCTGCCTG
	Reverse	GCTGCTCCACGAACTCAAACACCG
HMGCR	Forward	GTCATTCCAGCCAAGGTTGT
	Reverse	GGGACCACTTGCTTCCATTA
AR	Forward	GCCTTGCTCTCTAGCCTCAA
	Reverse	GTCGTCCACGTGTAAGTTGC
18S rRNA	Forward	GCTTAATTTGACTCAACACGGGA
	Reverse	AGCTATCAATCTGTCAATCCTGTC
GAPDH	Forward	GACAACAGCCTCAAGATCATCAG
	Reverse	ATGGCATGGACTGTGGTCATGAG
